# Knowledge and attitude toward genetic diseases and genetic tests among pre-marriage individuals: A cross-sectional study in northern Iran

**DOI:** 10.18502/ijrm.v17i8.4819

**Published:** 2019-09-03

**Authors:** Mohammad Bagher Hashemi-Soteh, Ali Vali Nejad, Golamreza Ataei, Alireza Tafazoli, Dariush Ghasemi, Rita Siamy

**Affiliations:** ^1^Immunogenetic Research Center, Molecular and Cell Biology Research Center, Medical Faculty, Mazandaran University of Medical Sciences SariMazandaran Iran.; ^2^Ghaemshahr Health Center, Mazandaran University of Medical Sciences Mazandaran Iran.; ^3^Department of Medical Physics, Faculty of Paramedicine, Babol University of Medical Sciences Babol Iran.; ^4^Department of Biochemistry, Biophysics and Genetics, School of Medicine, Mazandaran University of Medical Sciences Mazandaran Iran.; ^5^Department of Biostatistics, Faculty of Healthcare Sciences, Mazandaran University of Medical Sciences Mazandaran Iran.

**Keywords:** Attitude, Knowledge, Genetic testing, Genetic counseling, Prenataldiagnosis

## Abstract

**Background:**

Genetic testing has been widely introduced for many hereditary disorders. While the attitudes towards these facilities have been evaluated in many countries, there are only a few reports on the knowledge of and the orientation among Iranians.

**Objective:**

The current study assesses the attitudes and knowledge of pre-marriage individuals toward the availability and use of genetic tests.

**Materials and Methods:**

A comprehensive questionnaire was distributed among 408 marrying individuals. The questions addressed the demographic characteristics along the registration of participant's knowledge, education, and attitude toward genetic testing. The individuals were divided into three groups based on their knowledge: 1) Scored above 80 to 100 were defined as "good" 2) 60 to 80 as "average" 3) less than 60 as "poor" knowledge.

**Results:**

Most participants (86%) believed consanguineous marriages increase the risk of genetic diseases; 82.3% knew that thalassemia is a type of genetic disease, only 33.3% could distinguish prenatal diagnosis (PND) from other laboratory tests. The relationship between the participants' knowledge and their level of education was significant (r░=░0.78, p░<░0.001), age (r= -0.16, p░<░0.01), and urbanity (p░<░0.01). A prominent relationship was observed between the knowledge (r░=░0.64, p░<░0.001) or education (r░=░0.62, p░<░0.001) and people’s desire to use the genetic tests before the wedding ceremony. No significant correlations were found between the participant’s attitude and their ages/urbanity. Most of the individuals agreed to arrange a genetic counseling before marriage (0.94%).

**Conclusion:**

This study revealed that most individuals were interested in using genetic counseling services and genetic tests before marriage.

## 1 Introduction

The advances in DNA technology and chromosomal analysis methods during the past decadehave improved our understanding of genetic diseases and provide practical applications such as genetic testing and prenatal diagnosis (PND). In the era of next-generation sequencing (NGS) technologywith a vast range of application that provides an analysis of genes toany genetic disease in family, the knowledgeand attitude of people about genetic services areimportant (1). Also, precise PND results are available for several disorders that show significant morbidity and mortality in early life (2). Healthcare providers imply to use these facilities before marriage as a beneficial option. It is clear that genetic testing will be applied more commonly in medicine in the near future. People can benefit from genetic tests significantlyandcan help them make appropriate decisions about having a healthy child or managing their healthcare (3, 4). It is predictable that individuals with different levels of education or science literacy will have various level of understanding and issues of concern regarding genetic testing, so their approach might be significantly different (2, 5, 6). It appears that public attitude toward genetic testing among Europeans and US citizens is positive, and these kinds of tests are already used for common inherited diseases such as cystic fibrosis and thalassemia (7–11).

The majority of the Iranian population is Muslim with a high frequency of consanguineous marriages, and it is assumed that this will lead to an increased frequency in homozygosity and genetic disorders (12). Mazandaran, the Northern Province of Iran, with approximately 3 million populations, shows a high prevalence of thalassemia with almost 10% carriers (13, 14). PND has been available for thalassemia for more than a decade and testing for other genetic disorders is now established in the country (15, 16).

The aim of this study was to assess the knowledge and attitudes of pre-marriage individuals in northern Iran toward genetic diseases and genetic testing.

## 2 Materials and Methods

This study was a cross-sectional survey. This investigation used a prepared questionnaire and applied a descriptive study on premarital couples who were referred to the Health Care Center in Ghaemshahr, Mazandaran province, north of Iran. People were first asked if they are interested voluntarily to participate in this research (inclusion criteria), and there was no limitation of age, sex, race, education, or place to live, etc. (exclusion criteria); 88.8% of the volunteer individuals were from Mazandaran and 11.2% of them represented other ethnic groups living in Iran.

The sampling method was accessible as a kind of non-probability sampling approach. The Cochran ratio formula was utilized for estimating the sample size. The sample size was calculated as 384 individuals with p░=░0.5, d░=░0.05, and 95% confidence level;408 questionnaires were filled out by the volunteers. *Cronbach's alpha* was measured for the internal consistency of the questionnaire. The value of this index was 0.79 as an acceptable range for consistency for 408 individuals. The construct validity of the questionnaire was determined via factor analysis. The value of KMO index was 0.659, and it represented that sample size was adequate for factor analysis. Also,Bartlett’s Mauchly's*sphericity*test was rejected under alpha 0.05 (p░<░0.05). Questions covered demographic information such as: gender, ethnicity, location, employment, and educational status. Other questions were designed to evaluate the participant’s knowledge regarding genetic diseases like thalassemia (the most prevalent genetic disorder in the north of Iran), consanguineous marriage risks,and PND.They were invited to answer the questions about their attitude toward genetic counseling, consanguineous marriages, abortion, and religious limitations.

### 2.1 Ethical consideration

This study was approved by the ethics committee of Mazandaran University of Medical Sciences (IR.MAZUMS.REC.95.8623) and the procedures followed were in accordance with the ethical standards and in line with the Helsinki Declaration of 1975, as revised in 2008. All the participants have signed consent forms for participating in this project.

### 2.2 Statistical analysis

The statistical analyses were done using R software version 3.2 (free open source software, developed by cooperation between different countries: https://www.R-project.org/) (17). The relationship between continuous variables was considered using the Spearman Correlation test. Also, ANOVA with Tukeypost-hoc testandone-sample *t*-test wereperformed to assess the potential association between categorical and continues variables. The Kolmogorov-Smirnov test was performed for meeting the normality presumption, while the categorical variables were compared using the chi-squared test, as appropriate. All the p-values were estimated with statistical significance defied asa p░≤░0.05 and ≤ 0.001.

## 3 Results

### 3.1 Descriptive Statistics

There were 408 individuals, including 402 volunteers - 231 females and 171 males;64% of the participants were living in urban areasand 36% inthe suburbs or nearby villages. The majority of the participants werefrom the Mazaniethnic group, and the remaining were from Turkish, Kurdish and Gilaniethnics (18). The ages of individuals were between 15 and 43░yr with an average of 24░yr.

### 3.2 Knowledge of genetic disease and genetic testing

Individuals were divided into three groups based on their knowledge: 1) those whoscored above 80 to 100 were defined as "good"; 2) above 60 to 80 as "average"; 3) and those less than 60 as "poor"knowledge(Table I).

**Table I:** Correlation of the knowledge of volunteers and their attitudes toward genetic testing (p░<░0.001)

Attribute	Level	Total	Positive	Neutral	Negative
Knowledge	Good	96	39 (40)	46 (47.9)	11 (12.1)
	Average	205	50 (24.3)	103 (50.2)	52 (25.3)
	Poor	107	11 (10.3)	50 (46.7)	46 (43)
	Total	408	100	199	109
Education	Universities	86	36 (42)	32 (37.2)	18 (21)
	High school	211	43 (19.9)	115 (55.3)	53 (24.8)
	Lower	111	22 (20)	51 (46)	38 (34)
	Total	408	99	197	107

Data presented as n (%)

This study showed that while 23.5% of the participant had good knowledge about genetic diseases and testing (score 80 to 100), 50.2% had an average knowledge (score 60 to 80) and 26.3% had poor knowledge (score less than 60) (Table I). Most of the participants (86%) thought that consanguineous marriages increase the risk of genetic diseases in children,82.3% knew that thalassemia is a type of genetic disease, but only 33.3% could distinguish PND from other kinds of laboratory tests. There was a significant relationship between the knowledge and education (r░=░0.384, p░<░0.001), knowledge and age (r= -0.156, p░<░0.01), knowledge and career (p░<░0.01), and knowledge and urbanity (p░<░0.01), but there wasnosignificant association between the knowledge and gender (p░=░0.115) and knowledge and nation (p░=░0.698). Almost 59.3% of highly educated people with bachelor or higher university degree showed a well-understood information about genetic testing, while only 10.8% of the people who didn’t have a high school degree were among the group with good knowledgeabout genetic disease and tests. Among younger individuals who were between 15 and 20 yr old, only 8% demonstrated fine awareness about genetic diseases. On the other hand, young participants were moreaware of genetic disorders in comparison with their elder counterparts. People who resided in the countryside were much aware of genetic illnesses compared to urban citizens. In addition,among the participants, working employees had the most awareness, whereas farmers and labors had the least knowledge concerning genetic tests.

### 3.3 Attitude toward genetic diseases and genetic testing

To evaluate the attitude, participants were divided into three groups based on their scores: “positive” (30–36), “neutral” (25–30), and “negative” attitude (< 25). A strong relationship was achieved between the individuals’ attitude toward genetic testing and their level of knowledge (p░<░0.001) or education (p░<░0.001) (Tables I and II). Interesting results were achieved between the awareness and the attitude of the volunteers. About 40% of the attendants who previously had good knowledge represented positive attitudes, but 43% without previous knowledge had a negative trendtoward genetic testing. On average, about 48% of participants in all groups were neutral, regardless of their knowledge. The differences between negative trends toward genetic testing among high- andlow educated people were also significant with 21% and 34%, respectively. Meanwhile, no significant correlations were found between the participants’ attitudes and their ages or urbanity. There was a considerable relationship between the propensity and gender:29% of the females had a positive while 20% had negative tendencies toward genetic testing compared tothe males with 18% positive and 36% negative (p░<░0.01), respectively.

### 3.4 Attitude toward consanguineous marriages and genetic counseling

No significant differences were found between men and women's attitude toward consanguineous marriages (p░=░0.069). Also,their knowledge had no effect on their attitudes (p░=░0.090). The majority of the participantsbelieved that consanguineous marriages will hold risks for genetic disease in the next generation. While most of the entirelyagreed that couples should be counseled prior to their marriage, only 0.5% were in complete disagreement with receiving these services. The overall opinion of the participants was positive about the main items in questionnaire such as avoiding consanguineous marriages (86%), using genetic counseling before marriages (94%), and termination of the pregnancy in case of embryonic genetic disorders (73.2%).

## 4 Discussion

This study showed that 23.5% of the participants had good, 50.2% had average,and 26.3% of the population had poor knowledge about genetic diseases and testing. Most of the participants (86%) thought that consanguineous marriages increase the risk of genetic diseases in children. There was a significant relationship between participants' knowledge and their level of education, age, and urbanity. A strong relationship was achieved between individuals’ attitude toward genetic testing and their level of knowledge or education. About 40% of attendants who previously had good knowledge represented positive attitudes, but 43% without previous knowledge had a negative trend toward genetic testing. On average, about 48% of participants in all groups were neutral, regardless of their knowledge toward genetic testing.

People with different ethnicities have been living in Iran for a long time and this has made Iran's population a highly mixed one (19). Like the other Middle Eastern countries, consanguineous marriages are quite prevalent in Iran, and the mean percentage estimated is about 38% (12, 20). A national prevention program for thalassemia with screening, counseling, and PND networks was developed in 1997 in Iran (15, 21).

There was a significant decrease in consanguinity among high-risk thalassemia families in Iran since the national thalassemia program was established (16, 22). This major reduction appears as a result of increased awareness and tends to have a premarital genetic counseling session among the public about thalassemia (21).

Among the individuals with bachelor or higher university degree, 42% showed well attitude and perception about the genetic tests, while people with high school degrees only showed 20% attitude in this regard (Table I). Similar results were shown when senior college students were asked about genetic testing in Saudi Arabia. The majority of students showed positive attitudes toward genetic testing, although some students had negative attitudes toward abortion of an untreatable major genetic defect (23). The results of this survey have shown that: firstly, the younger the people are, the more they know about genetic disorders; secondly, the higher the level of education, the better awareness about genetic diseases;and lastly, the tougher the career is, the less knowledge about genetics exists. However, there is a common belief that perhaps people residing in the rural areas would not be as aware as the urban citizens about the scientific topics such as genetics; this study depicted the opposite. In other word, countryside people was more aware about genetic tests than the urban citizens as achieved higher scores in this study. In spite of these findings, it seems that neither being a male or a female nor nation’s diversity can change the level of knowledge regarding genetic illnesses (Table II). One of the major concerns was the attitudetowardthe pregnancy termination. About 50% believed that abortion for genetic disorders is unacceptable; however, 73.2% of them said that they would terminate the pregnancy if their child had a serious genetic problem. This ambiguity in public attitude for genetic testing has been demonstrated earlier in Finnish people (24). The results of the Finland survey showed that only 6% of the participants believed that they will have healthy children without genetic testing and these tests are unnecessary, while 94% of them agreed with genetic counseling. Roberts and colleagues reported that among 87% of women who were referred to genetic counseling by their physicians, 65% said they would abort their fetus if it had any disorders and 74% found genetic counselors as a source to help them to decide (25). Another study, an online survey in Italy,investigated knowledge about genetic risk, genetic screening, and personal attitudes toward genetic testing. Results revealed that although participantsbelieved that genetic assessment is helpful for disease prevention, about 67%of them had never heard about genetic testing directly available to the public, and they thought it could affect their life planning with little clinical utility (26).

**Table II:** Statistical tests to assess a potential relationship between demographic traits and individual's knowledge and attitude toward the genetic tests

Attribute	Category	Knowledge mean (in percentage)	F/T	P-value	Attitude mean	F/T	P-value
Sex	Female	67.67	-1.571	0.117	2.83	3.269	0.001^**^
	male	65.05			3.02		
Education	Under diploma	57.73	49.246	0.000^**^	3.04	5.836	0.003^**^
	diploma and post diploma	66.13			2.91		
	BSc and MSc	79.07			2.75		
Career	Employee	72.55	3.732	0.005^**^	2.92	2.460	0.045*
	labor	59.71			3.04		
	farmer	51.28			2.93		
	self-employed	64.22			3.03		
	others	67.41			2.83		
Nation	Mazani	66.38	-0.660	0.509	2.91	0.323	0.747
	non-mazani	68.09			2.88		
Urbanity	Rural	68.58	3.033	0.003^**^	2.89	-0.680	0.497
	urban	63.33			2.93		

** Significant at the level of 0.01; *Significant at the level of 0.05

The relationship of continuous variables was considered using the Spearman Correlation test.

ANOVA with Tukey post hoc and one-sample *t*-test were performed to assess the between the categorical and continues variables.

Results achieved in this study also showed that 73.2% of women said they would terminate the pregnancy if their fetus borea genetic disorder. Another study in Iran revealed that 91.3% of physicians support abortion in the case of thalassemia (27). Also, there is religious legal permission for abortion (Fatwa) before the 19th week of pregnancy in Iran. The Iranian parliament approved a new act of abortion in2005. Prenatal screening as a compulsory practice mandated by law is offered in several states of the United States of America.Also, in the United Kingdom, it is being recommended to the government by the healthcare providers that screening could be offered to all pregnant women regardless of their ages (2, 28).

## 5 Conclusion

The results of this study revealed that most of the participants were interested in using genetic counseling services and genetic tests, so these kinds of services such as PND should be available for everyone. It is evident that professional teams with proficiency in genetics and interpersonal communications are required for increasing the awareness of society in genetic fields, which lead to informed decision-making, especially for parents who intend to have a child.

## Conflict of Interest

The authors report no conflicts of interest.
